# Immunopotentiating and Delivery Systems for HCV Vaccines

**DOI:** 10.3390/v13060981

**Published:** 2021-05-25

**Authors:** Alexander K. Andrianov, Thomas R. Fuerst

**Affiliations:** 1 Institute for Bioscience and Biotechnology Research, University of Maryland, Rockville, MD 20850, USA; tfuerst@umd.edu; 2 Department of Cell Biology and Molecular Genetics, University of Maryland, College Park, MD 20742, USA

**Keywords:** immunoadjuvants, hepatitis C virus (HCV), vaccines, delivery systems, immunopotentiation, pharmaceutical formulations, self-assembly, multimericity

## Abstract

Development of preventive vaccines against hepatitis C virus (HCV) remains one of the main strategies in achieving global elimination of the disease. The effort is focused on the quest for vaccines capable of inducing protective cross-neutralizing humoral and cellular immune responses, which in turn dictate the need for rationally designed cross-genotype vaccine antigens and potent immunoadjuvants systems. This review provides an assessment of the current state of knowledge on immunopotentiating compounds and vaccine delivery systems capable of enhancing HCV antigen-specific immune responses, while focusing on the synergy and interplay of two modalities. Structural, physico-chemical, and biophysical features of these systems are discussed in conjunction with the analysis of their in vivo performance. Extreme genetic diversity of HCV-a well-known hurdle in the development of an HCV vaccine, may also present a challenge in a search for an effective immunoadjuvant, as the effort necessitates systematic and comparative screening of rationally designed antigenic constructs. The progress may be accelerated if the preference is given to well-defined molecular immunoadjuvants with greater formulation flexibility and adaptability, including those capable of spontaneous self-assembly behavior, while maintaining their robust immunopotentiating and delivery capabilities.

## 1. Introduction

Development of a preventive vaccine against hepatitis C virus (HCV), which affects an estimated 71 million people globally [1], is considered to be one of the main strategies in eliminating the disease and reducing the public health burden [2]. However, despite decades of research, there is currently no licensed vaccine against HCV [1,3]. This has been attributed to a number of challenges. The extreme genetic diversity of HCV resulting from a high rate of virus mutation requires a broad immune response to conserved regions capable of reacting to abundant variations in viral species [4]. Despite this extraordinary diversity, some antibodies, referred to as broadly neutralizing antibodies (bNAbs), recognize relatively conserved regions in the HCV envelope glycoproteins, E1E2, and block infection by genetically diverse HCV isolates [5,6]. Early development of bNAbs is associated with natural control of HCV infection in humans, and bNAbs can prevent HCV infection in animal models. Therefore, an effective HCV vaccine will almost certainly need to induce bNAbs [6,7,8,9]. Moreover, induction of protective responses requires maximizing T-cell and antibody responses, which in the case of relatively weakly immunogenic HCV antigens, dictates the need for potent adjuvant and delivery mechanisms [5,10,11].

Generation of robust immune responses by subunit vaccines has become increasingly dependent on the aid of potent immunoadjuvants. These additives, which are designed to enhance, prolong, and modulate antigen-specific responses include “true” immunopotentiating compounds, as well as vaccine delivery vehicles [12,13,14,15,16,17]. The most widely used immunoadjuvant—Alhydrogel (Alum)—has been recently supplemented with a number of advanced systems, such as MF59, AS03, AF03, AS01, and AS04, which are now employed in licensed vaccines [16,17]. Much progress has been made in the identification of cellular targets for immunopotentiating molecules, including those employed with HCV antigens [10,18,19,20,21]. Nevertheless, the search for a safe and efficient adjuvant continues and the most recent and advanced immunoadjuvant systems appear to exploit the synergy between delivery and immunopotentiation mechanisms [16].

This review provides an analysis of current information on both immunopotentiating compounds and vaccine delivery systems, which have been studied with HCV antigens, with a focus on the interplay between the two main modalities. It details structural, biophysical, and mechanistic features of immunoadjuvants with the goal of evaluating potentials for their synergy and their suitability for use with HCV antigens. Finally, the review summarizes the main outcomes of recent preclinical and clinical studies and discusses potential pathways for the development of successful adjuvants for HCV vaccines.

## 2. Immunoadjuvants Investigated in Formulations with HCV Antigens

Classification of adjuvants on the basis of their mechanism of action is never absolute, as several mechanisms frequently co-exist and most advanced adjuvant formulations tend to combine, or even integrate, immunopotentiating molecules and delivery vehicles in the same entity [22]. Adjuvants used in formulations with HCV antigens are nevertheless conditionally can be grouped as below on the basis of their prevailing or most fully investigated functionality.

### 2.1. Adjuvant Formulations with Prevailing Delivery Functionality

**Figure 1:**
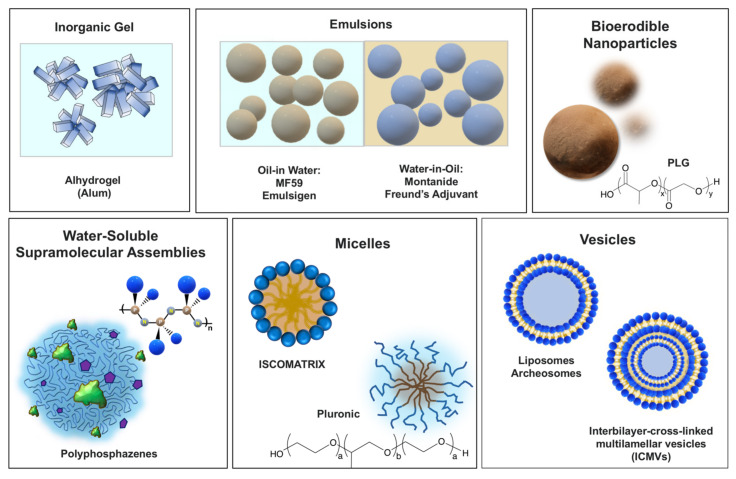
Vaccine adjuvants with predominant delivery function grouped based on their physico-chemical features.

Vaccine delivery vehicles typically include dispersed systems in which an antigen is either encapsulated or surface adsorbed. These adjuvants generally function by promoting the uptake of vaccine antigens into the immunocompetent cells. Their main classes are schematically presented in Figure 1.

#### 2.1.1. Inorganic Gels

*Alhydrogel (Alum)*. Particulate aluminum hydroxide (sometimes aluminum phosphate), which is commonly referred to as Alhydrogel or Alum, has been used in clinics for decades and is licensed for multiple vaccines [23]. Its role as a delivery vehicle has been long accepted. Adsorption of antigens on the surface of Alhydrogel particles facilitate interactions with antigen-presenting cells, which is also commonly described as the formation of ‘antigen depot’ at the injection site [23]. In particular, the delivery function of Alhydrogel can be illustrated by the data on the uptake by DC in vitro, which is dependent on the size of antigen-Alhydrogel aggregates [23]. However, recent studies also suggest multiple mechanisms of adjuvant activity. In particular, it was reported that in human and mouse macrophages, Alhydrogel activates inflammasome, which is mediated by the NLR (nucleotide-binding domain leucine-rich repeat-containing) protein NLRP3 [24,25]. It is worth noting that Nlrp3 inflammasome is also activated by other particulate compounds and the contribution of molecular and cellular events to adjuvanticity of Alhydrogel is still under discussion [23]. Most recently, aluminum salts have been formulated with immunopotentiator, MPL (Section 2.2.1), to produce AS04 adjuvant, which is approved for licensed hepatitis B and human papillomavirus vaccines [14,16].

#### 2.1.2. Emulsions

Emulsions also have a long history in vaccine formulations and can be broadly divided on the basis of inner/outer phase into water-in-oil and oil-in-water systems.

*Freund’s adjuvant.* “Incomplete Form” of Freund’s adjuvant (IFA) is a water-in-mineral oil emulsion, whereas “Complete Form” (CFA) also contains inactivated and dried mycobacterial cells. Both adjuvants have been used in research for almost eighty years, but adverse reactions prevented their licensure for human or veterinary use [26].

*MF59*. Oil-in-water emulsion adjuvant based on biodegradable squalene oil (droplet size—160 nm), which was approved for use in human vaccines in 1997 [17]. MF59 activates dendritic cells (DCs) and macrophages in the muscle, inducing a mixture of chemokines, which results in the migration of immune cells into the injection site and efficient transport of antigen to the lymph nodes [27]. Importantly, none of the individual components of the emulsion are an adjuvant and association of antigens with the droplets are noted to be beneficial for the adjuvant effect confirming their delivery modality [17]. AddaVax is another squalene-based oil-in-water nano-emulsion, which is claimed to be similar to MF59 [28].

*Montanide ISA*. A clinical-stage adjuvant based on water-in-mineral oil emulsion, which is stabilized with a surfactant from the mannide monooleate family [29]. It enhances antigen-specific antibody titers and cytotoxic T-lymphocyte (CTL) responses and the activity is reported to be associated with depot formation, inflammation and lymphocyte-trapping [29]. However, serious adverse effects and premature termination of two clinical trials with Montanide ISA 51 advised caution in its further clinical development [29].

#### 2.1.3. Bioerodible Polymer Micro- and Nanoparticles

*PLG particulates.* Particulate systems on the basis of biodegradable water-insoluble polymer-poly(lactide-co-glycolide) (PLG), is the most common representative of this class and has been used in a number of licensed biopharmaceutical products requiring modulated release [30]. PLG micro- and nanoparticles have been also extensively studied as a vaccine delivery carrier. However, encapsulation of antigenic proteins in this hydrophobic polymer has been proved exceptionally challenging due to the use of organic solvents and acidic environment and even judged to be a “mission impossible” [17]. Alternatively, antigen can be adsorbed on the surface of microparticles with adjuvant potency results either comparable or only marginally superior to those of Alhydrogel or emulsions [30]. The system may permit a synergistic co-delivery of surface adsorbed antigen and immunopotentiators encapsulated in polymer matrix [30]. One of the technological challenges introduced by high moisture sensitivity of the polymer, is that the particles need to be lyophilized and redispersed with antigen formulation before administration [17].

#### 2.1.4. Water-Soluble Self-Assembling Supramolecular Systems

*Polyphosphazenes.* This class of delivery system with some immunopotentiating capability is mainly represented by a family of biodegradable organic-inorganic macromolecules-polyphosphazenes [31]. These water-soluble macromolecules can simultaneously bind and effectively display antigens and immunostimulating molecules, such as resiquimod (R848), through spontaneous supramolecular assembly process in aqueous solutions [32,33]. The resulting assemblies resemble viruses in terms of their dimensions (60–80 nm) and multimeric co-presentation of antigens and “danger signals”. Poly[di(carboxylatophenoxy)phosphazene], PCPP-a clinical stage macromolecule, along with its structural homologs-PCMP and PCEP have been shown to display high potency with multiple viral and bacterial antigens [31]. An important feature of these systems is their ability to stabilize vaccine antigens in solution [34,35,36].

#### 2.1.5. Micelles

*Pluronics.* Pluronics, or polyoxamers, are non-ionic, amphiphilic block copolymers of polyoxyethylene and polyoxypropylene (POE–POP), which form micelles in aqueous solutions, and have been extensively studied for gene delivery applications [37]. Although the mechanism of adjuvant activity is not yet elucidated, the effect is believed to be associated with sustained antigen delivery [38]. It was also noted that, at least in some cases, these polymers can activate selected signaling pathways, such as NF-κB [37].

*ISCOMATRIX.* The term ISCOMATRIX adjuvant is derived from “immune stimulating complexes” (ISCOMs). The adjuvant is composed of cholesterol, phospholipid, and saponin, which form spherical micellar assemblies upon mixing [39,40]. The diameter of ISCOMATRIX particles, which is originally about 40 nm [39], can be greatly affected upon formulation with the antigen and can eventually increase to microns [41]. The immunoadjuvant activity of ISCOMATRIX adjuvant is due to the combination of antigen presentation and immunomodulatory activity of the saponin component (Section 2.2.4) [40].

#### 2.1.6. Vesicles

*Liposomes and other nanovesicles.* Liposomes, archaeosomes, virosomes, and other nanovesicles containing one or multiple lipid bilayers have become important delivery systems in vaccine development [42]. In these systems, antigen and adjuvant can be entrapped within the aqueous inner space, intercalated into the lipid bilayer or attached either adsorbed or attached to the liposome surface covalently [42]. Although the system is versatile in terms of surface chemistry and dimensions, liposomes frequently require stabilizers. Interbilayer crosslinked multilamellar vesicles (ICMVs), have the potential to improve encapsulation and stability over the more common multilamellar liposomal vesicles [17,42].

*Liposome-based combination adjuvants.* One important example of advanced liposome-based vaccine adjuvant system is AS01, which also contains containing two immunostimulants: 3-O-desacyl-4′-monophosphoryl lipid A (MPL) and saponin-based QS-21 [43]. Another-CAF01 is a cationic adjuvant formulation consisting of dimethyldioctadecylammonium liposomes as a delivery vehicle and synthetic mycobacterial cordfactor as immunomodulator [44]. Adjuplex is a research adjuvant, which combines a copolymer of poly(acrylic acid) (Carbopol) and submicron-sized liposomes derived from purified soy lecithin [45].

### 2.2. Adjuvants Functioning as Immune Potentiators

**Figure 2:**
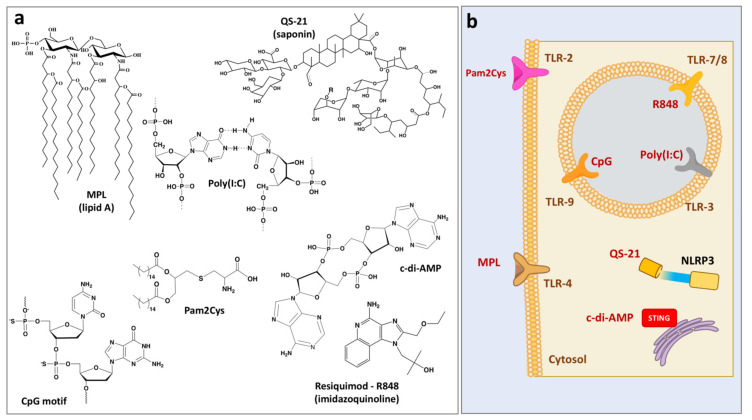
(**a**) Chemical structures of immunostimulating molecules and (**b**) their cellular targets.

In contrast to vaccine delivery systems, immunopotentiators augment immune responses not through facilitating the uptake of the antigen, but mainly by direct activation of immune cells. These biologically active compounds (Figure 2a) either constitute or mimic components of pathogens and recognition of their molecular structures is usually mediated by pattern recognition receptors, which include Toll-like receptors (TLRs), NOD-like receptors and others (Figure 2b) [17,46,47]. Innate immune cells perceive these compounds as “danger signals” and the resulting activation is characterized by the production of proinflammatory cytokines, chemokines, and antimicrobial peptides [46].

#### 2.2.1. MPL

Monophosphoryl lipid A (MPL) adjuvant is a chemically detoxified derivative of native Lipid A from *Salmonella minnesota* R595. This TLR4 ligand promotes generation of Th1 responses through the release of proinflammatory cytokines (TNF, IL-2 and IFN-gamma) and has proven to be safe and effective in inducing immune responses to antigenic proteins in animal and human vaccines [14]. MPL is part of licensed or clinical phase adjuvants, which are frequently used with delivery systems, such as liposomes and Alhydrogel: AS01 (MPL/QS21/liposomes), ASO2 (MPL/QS21/emulsion), AS04 (MPL/aluminum salt), and AS15 (MPL/QS21/CpG/liposomes) [14,16].

#### 2.2.2. CpG

Synthetic oligodeoxynucleotides (ODNs) containing unmethylated Cytosine and Guanine deoxynucleotides linked with phosphodiester (CpG) motifs target immune cells expressing TLR9 receptors boosting humoral and cellular antigen-specific immune responses [46]. CpG 1018 adjuvant-a short (22-mer) oligonucleotide sequence containing CpG motifs [48], is part of FDA approved hepatitis B vaccine [49].

#### 2.2.3. Resiquimod (R848)

Resiquimod (R-848) is a member of imidazoquinoline family of immunopotentiators, which activate immune responses in TLR7/8 dependent mechanism and possess strong anti-viral and anti-tumor activity [50,51]. R848 induces cytokine secretion, macrophage activation and enhancement of cellular immunity and is a clinical stage adjuvant [52,53,54]. However, utility of this molecule as vaccine adjuvant is reportedly impeded by short half-life and rapid dissociation from the antigen upon injection [55], which can be potentially overcome by its association with polyphosphazene adjuvant as a counterion (Section 2.1.4), or through its inclusion into various micro- and nanoparticulate formulations.

#### 2.2.4. QS-21-Saponin-Based Adjuvant

QS-21 is a structurally defined saponin (triterpene glycoside), which is derived from the bark of the *Quillaja saponaria* Molina tree [56]. The suggested mechanism of action includes activation of the intracellular nucleotide-binding oligomerization domain-like (NOD-like) receptor (NLRP3) inflammasome with release of cytokines, which are important for the induction of Th1 response [57,58]. As mentioned in Section 2.2.1, QS-21 is part of AS01 and AS02 combination adjuvant systems and is frequently used with delivery systems, such as liposomes or emulsions. ISCOMATRIX (Section 2.1.5) is another adjuvant system, which may contain either QS-21 or another saponin-Quil A.

#### 2.2.5. Poly(I:C)

Polyinosinic:polycytidylic acid-Poly(I:C) is synthetic double-stranded RNA is known to interact with endosomal TLR 3 [59]. It induces inflammatory cytokine and chemokine production, type I interferon (IFN) in particular, and facilitates dendritic cell maturation [59].

#### 2.2.6. Pam2Cys

S-[2,3-bis(palmitoyloxy)propyl]cysteine (Pam2Cys), is a lipopeptide, which activates TLR-2 and shows promise as vaccine adjuvant candidate [60]. Derivatives have been synthesized, such as anionic E_8_Pam_2_Cys [61].

#### 2.2.7. c-di-AMP

The cyclic di-nucleotide bis-(3′,5′)-cyclic dimeric adenosine monophosphate (c-di-AMP) is a promising adjuvant for parenteral and mucosal immunization [62,63]. It is an efficient activator of STING (stimulator of interferon genes)—an endoplasmic reticulum adaptor that facilitates innate immune signaling [64].

## 3. In Vivo Studies Using Protein-Based Vaccine Candidates

Research efforts on the development of HCV vaccine have been largely focused on the search for efficient immunogen and side-by-side comparison of adjuvants is relatively uncommon. Table 1 provides a brief summary of adjuvanted protein-based formulations that have been studied in various animal models. Alhydrogel, along with various emulsion-based systems, remain some of the most popular choices. Nevertheless, a number of studies report their inferior performance compared to other adjuvants used, unless the formers are combined with potent immunostimulants, such as TLR agonists.

In one of the most inclusive HCV-related investigations of immunoadjuvants, the authors compared emulsion (MF59), lipid-based nanoparticles (archaeosomes), and combination of delivery system-immunostimulant: Alhydrogel-MPL [73]. The study was conducted in mice using recombinant HCV glycoproteins E1E2. All adjuvanted formulations showed improved immunogenicity with significant neutralization activity compared to the antigen alone; however, a cellular response was not detected for MF59 adjuvanted formulation [73]. In another study, MF59 alone also was potent in inducing higher neutralizing titers to E1E2, but did not induce cellular immunity [78]. However, combination of this oil-in-water emulsion with immunostimulant, CpG, resulted in potent CD4^+^ or CD8^+^ T cellular immune responses [78]. It was also reported that T cell response observed for E1E2 formulated in archaeosome was superior to the same antigen adjuvanted with Alum-MPL [73].

Comparison of immunoadjuvant activity of MF59, Alhydrogel, and PLGA microparticles was also conducted using polyprotein comprising core and non-structural NS3, NS4a, NS4b, NS5a, and NS5b proteins [79]. MF59 and PLGA formulations induced similar and stronger serum IgG titers and Alhydrogel was found to be the least effective adjuvant. The trend was maintained also for T-cell proliferative responses. Interestingly, the addition of CpG to the studied delivery systems in this case did not enhance the proliferative response further, except for the Alhydrogel group.

MPL, TLR4 agonist, is another adjuvant, which has been widely investigated with E2, E1E2, VLPs, and other HCV antigens. It is commonly formulated with various delivery systems: encapsulated in liposomes with saponin-based QS-21 (AS01B) [83,84], in interbilayer-cross-linked multilamellar vesicles (ICMVs) [65], with Alhydrogel [73], trehalose [91], or adsorbed on the surface of nanocapsules composed of poly((ethylene-co-butylene)-b-(ethylene oxide)) [81]. In the form of AS01B, it promoted four-fold increase in antibody titers to HCV VLPs in mice and shifted the response towards desirable Th1 immunity [83]. The effect of CpG-TLR7/8 agonist, was similar (3-fold increase), and the combination of both adjuvants was synergistic leading to ten-fold increase in anti-E1/E2 antibody responses. However, it was also reported by the same group that immunogenicity of HCV-VLPs in baboons was only marginally increased by AS01B or their combination with CpG [84]. These results emphasize limitations of the mouse model in conducting HCV vaccine adjuvant research.

Particulate saponin-based adjuvant, ISCOMATRIX, was shown to be an effective adjuvant promoting cellular and humoral immune responses in nonhuman primates when formulated with core or E1E2 antigens [41]. However, authors also note that adsorption of core antigen on this adjuvant resulted in aggregates, which were approximately twenty-five-fold larger than ISCOM (1 mm vs. 40 nm, correspondingly). Combination of ISCOMATRIX with CpG was found to be effective in inducing Th1-type CD4^+^ T-cell responses with NS345core polyprotein in mice [78].

Formation of physiologically stable complexes between antigens and adjuvants is a rapidly evolving approach, which is characterized by simplicity of formulation and robust in vivo performance [31,61]. Spontaneous assembly of synthetic branched cationic or anionic lipopeptides, which contain TLR-2 agonist Pam2Cys, with HCV VLPs resulted in the formation of stable supramolecular complexes [85]. In mice, Pam2Cys adjuvanted VLPs demonstrated superior performance compared to non-adjuvanted or traditionally alum-adjuvanted VLPs both in terms of antigen-specific humoral and cell-mediated responses.

Polyphosphazene adjuvants represent an attractive immunoadjuvant and vaccine delivery platform, which is empowered by the spontaneous formation of non-covalent assemblies with HCV antigens. Biophysical features of these water-soluble synthetic macromolecules and their dimensions (60–120 nm) enable supramolecular assemblies, which not only mimic the size of the virus, but also permit binding of multiple copies of the antigen thereby supporting its multimeric presentation. For example, it was reported that poly[di(carboxylatophenoxy)phosphazene], PCPP, with a hydrodynamic diameter of 60 nm can present up to thirty-five molecules of HCV E2 antigen [32]. Furthermore, utilizing ionic interactions, the same polymer chain can bind and effectively retain under physiological conditions, numerous molecules of TLR7/8 agonist-resiquimod (R848). The resulting ternary virus-mimicking polymer assembly (VMPA), which simultaneously displays antigens and “danger signals” in their multimeric form, demonstrated high potency in inducing both humoral and cellular responses in mice [32]. Some structural alterations in polyphosphazene structure realized in more advanced derivatives, such as poly[di(carboxylatoethylphenoxy)phosphazene], PCEP, which already demonstrated superior in vivo performance with HCV antigens [66]. Due to utmost simplicity of spontaneous self-assembly and co-encapsulation of immunopotentiating molecules, which is achieved by simple mixing of water-soluble components, the system appears to be ideally suited for simultaneous testing of multiple antigens, as well as rapid screening of antigenic constructs designed through computational methods [74].

Mucosal immunization remains one of the most appealing alternative routes of vaccine administration. The use of mucosal immunoadjuvant, c-di-AMP, with HCV glycoproteins E1E2 in mice offered the advantage of inducing a superior cellular response compared to parenterally administered MPL-Alhydrogel or MF-59 adjuvant system [73]. The regimen comprising an intramuscular immunization followed by two intranasal boosts was most effective leading to the induction of both robust humoral and cellular immune responses. Given results for this adjuvant with other antigens, it was somewhat surprising that three intranasal administrations of c-di-AMP adjuvanted formulation did not induce neutralizing antibody titers, although still eliciting strong CD4^+^ T cell responses. Nevertheless, partial intranasal immunization regimen may still present some advantages in avoiding repeated intramuscular injections.

Overall, the results of recent studies indicate that adjuvantation of protein-based HCV vaccine candidates remains a challenging task. It is also apparent that both, animal model and route of administration, may have an additional and even unexpected impact on the study outcome. To add to the complexity, the analysis of in vivo studies in animals still presents a challenge. Typically used HCV pseudoparticles (HCVpp) or replication-competent cell culture viruses (HCVcc) to evaluate the serological breadth of neutralization only represent a small repertoire of the many polymorphisms present in naturally circulating HCV isolates, and may not address other confounding factors such as lipids associated with the native virion [94,95]. Nevertheless, the approaches that utilize a combination of an effective and formulation-simple delivery system with potent immunostimulant appear to hold a great deal of promise.

## 4. In Vivo Studies Using DNA-Based Antigens

Immunization with plasmid DNAs is an alternative strategy, which rapidly gains popularity and has been also explored for potential HCV vaccine applications. Despite multiple clinical trials of DNA vaccines, their immunogenicity in human remains low [96]. Due to the need for translocation of active cargo to the nucleus for transcription, its reliance on various delivery vehicles appears to be even greater than for protein-based vaccines. The role of DNA nanocarriers includes prevention of extracellular DNA degradation, enabling targeting of antigen presenting cells, and enhancing endo/lysosomal escape of DNA [96]. Delivery of DNA vaccines are frequently assisted by biophysical techniques, such as electroporation or gene electro-transfer (GET). Representative studies of HCV DNA vaccines are summarized in Table 2. As an example, the use of GET for intramuscular injection of a plasmid encoding HCV E2 glycoprotein resulted in a 10- to 30-fold enhancement in humoral responses in mice, rats, and rabbits compared to that induced by conventional naked DNA immunization [97]. Furthermore, GET assisted co-injection of E2 and cytokine (adjuvant) encoding plasmids strongly enhanced T- or B-cell responses [98]. In particular, by using various cytokine encoding plasmids authors were able to shift immunes responses towards its specific arms: antibody responses following IL-12 administration, CD4 following GM-CSF, and CD8 following IFN-α immunization. The use of nanoparticulate delivery vehicles is typically synergistic with electroporation. Mice immunized with electrically activated plasmonic gold nanoparticles showed up to 100-fold higher gene expression compared to control treatments (without nanoparticles) and exhibited significantly increased levels of both antibody and cellular immune responses against HCV DNA vaccine [99].

Biodegradable cationic PLGA microparticles have been reported as effective carriers for E1E2 encoding plasmid without the need for electroporation [71]. In mice, these formulations showed higher immune responses then those for naked DNA, provided for significant (10-fold) antigen dose sparing effect, and induced antibody titers comparable to those of MF59 adjuvanted recombinant E1E2 protein. Although, the latter was not achieved in non-human primate model, authors note that a single booster dose of recombinant protein administered to the animals previously immunized with microparticulate DNA formulation resulted in improved responses.

## 5. Adjuvants in Clinical Trials of HCV Vaccines

MF59 adjuvant was studied in two randomized, double-blinded, placebo controlled clinical trials with either HCV E1 and E2 glycoproteins [106] or HCV E1E2 [107]. Both studies utilized the same regimen: four immunizations using different doses of antigen-4, 20, or 100 µg in each group. Vaccines were reported to stimulate significant humoral [106,107] and cell-mediated [107] immune responses. Further testing of serum samples using well-characterized pseudoparticle (HCVpp) and cell culture replicating virus systems (HCVcc) demonstrated that immunization induced a cross-reactive neutralizing antibody response [108]. Interestingly, both studies did not observe statistically significant correlation between the level of antibody responses and the dose of immunogen. Authors noted that the absence of dose-response effect may be attributed to the dose sparing effect of MF59 adjuvant [107]. MF59 adjuvanted E1E2 vaccine was also evaluated in chronic hepatitis C patients treated with pegylated-interferon and Ribavirin and achieved sustained virological response [109]. Further analysis of the data obtained with MF59 adjuvanted HCV E1E2 vaccine indicated that cross-neutralization titers tended to be low and detected in only a minority of vaccinees [110]. Authors emphasize the need to enhance the immunogenicity of the vaccine by optimization of antigens, adjuvant, and their formulation [110].

Alhydrogel was investigated in clinical trials with twenty volunteers using intramuscular injection of adjuvanted HCV E1 formulation [111]. The selection of the adjuvant was driven by unpublished results in mice, in which Alhydrogel performed well in enhancing the induction of antibody titers and non-adjuvanted E1 itself induced high T-cell stimulation. Individuals who received the vaccine developed strong, specific cellular immune response towards E1, as well as humoral anti-E1 responses, although the neutralization capacity of the antibodies was not evaluated. Intramuscularly administered Alhydrogel adjuvanted formulation induced superior humoral and cellular immune response compared to subepidermal administration of unadjuvanted E1 [112].

Evaluation of ISCOMATRIX adjuvant was conducted in Phase I placebo controlled, dose escalation clinical study of the HCV Core based vaccine in thirty healthy individuals [113]. Out of subjects who received three immunizations, antibody responses were detected in all but one of the participants; however, the authors note no indication of dose response. CD8^+^ T cell responses were only detected in two of the eight participants receiving the highest dose.

Immunoadjuvant potency of poly-L-arginine was evaluated with a synthetic peptide vaccine containing HCV T-cell epitopes in healthy subjects [114]. The vaccine induced responses in all groups with the CD4^+^ T cell responder rate varying from 25 to 50% and CD8^+^ rate ranging from 0 to 42% depending on doses and number of vaccinations. Authors conclude that poly-L-arginine was required for the induction of Th1 polarized immunity. However, in the study of the same vaccine in chronic hepatitis C patients, authors could not establish dose response to poly-L-arginine and highlighted the need for more powerful adjuvants for peptide vaccines [115]. Additional study using the same vaccine, but administered via either subcutaneous or intradermal injections enhanced T cell responses up to two-fold [116]. Topical application of imiquimod to the site of injection reduced T cell response with intradermal injections causing more pronounced reactions, especially erythema and edema.

Recent clinical study of DNA vaccine with four plasmids encoding NS3/4A, NS4B, NS5A formulated with interferon lambda 3 (IFNL3) gene adjuvant was conducted in eighteen subjects [117]. Vaccine was administered as intramuscular injection followed immediately by electroporation with a series of four doses. Authors concluded that IFNL3-adjuvanted vaccine enhanced T cell responses and decreased regulatory T cell frequency in patients with chronic HCV infection.

## 6. Conclusions

The quest for immunoadjuvant system capable of effective co-delivery of antigen and immunopotentiator appears to be the most promising approach for vaccines in general. This can be achieved by the use of nanoparticulate delivery vehicles, such as nanosystems based on hydrophobic biodegradable polymers or liposomes. However, the challenge of synchronizing presentation of surface adsorbed antigen and release of immunopotentiating molecule, along with the need for preparing formulations of hydrolytically degradable polymers close to the time of administration or the necessity for additional efforts on stabilizing other dispersed systems, such as liposomes. appears to be substantial. To that end, the use of self-assembling systems capable of simultaneous multimeric presentation of antigen and immunopotentiating molecules, may be preferential to methods, perhaps efficient, but taking significant effort in formulation development.

One of the specific challenges in a search for immunoadjuvants, which are well-suited for HCV vaccines, lies in high variability of the virus, leading to the need for either combining multiple antigens or screening of computationally designed antigenic constructs. Analysis of the research undertaken in present review emphasizes difficulties in side-by-side comparison of different immunoadjuvants simply due to the diversity of HCV antigens. In general, it is well-established that models for adjuvanticity are not yet standardized and that the outcome of vaccination depends on multiple variables with those on the formulation side-doses, antigen purity and stability, intermolecular interactions, and presence of excipients-to be some of the most challenging. To that end, dealing with formulations also containing new and multiple immunogens in the timely fashion constitutes a significant hurdle. Alhydrogel presents a convenient choice from the formulation standpoint as it allows co-adsorption of multiple antigens, as well as addition of immunopotentiating molecules, as it was achieved in AS04. More sophisticated systems, such as liposomes and emulsions, although generally efficient, take a significantly larger effort to produce well-defined and stable systems. Still, all of these formulations, including Alhydrogel, are heterogeneous and are usually not straightforward in terms of their characterization and reproducibility. Water-soluble self-assembling systems, with polyphosphazenes as one example, appear to bring significant advantages when it concerns preparation of multi-component formulations or rapid screening of newly computationally designed construct. Water-soluble formulations allow for direct (without the need for antigen desorption) and comprehensive characterization using traditional suites of physico-chemical and biophysical methods. This combines with utmost formulation simplicity, compatibility with immunopotentiating molecules, stabilizing properties, biodegradability, and proven in vivo potency. All of these factors can potentially accelerate the progress in this challenging and significant area of the research.

## Figures and Tables

**Table 1:** Immunoadjuvants and vaccine delivery systems employed with protein-based HCV antigens.

Antigen	Adjuvants	Animal Model/Route ^1^	References
E2	MPL/ICMVs	Mice; s.c.	[65]
E2	PCPP, PCPP-R848	Mice; i.p.	[32]
E2	PCPP, PCEP	Mice; i.p.	[66,67]
E2	Addavax	Guinea pigs; s.c.	[68]
E1, E2	NP ^2^ (Fullerene)	Mice; s.c.	[69]
E1, E2	MF59, muramyl tripeptide	Chimpanzees; i.m.	[70]
E1E2	MF59	Mice; macaques	[71]
E1E2	Addavax, Adjuplex	Mice; macaques	[72]
E1E2	Alhydrogel-MPL, MF59, c-di-AMP, archaeosomes	Mice; i.m., i.v.	[73]
E1E2	PCPP-R848	Mice; i.p.	[74]
Core	Freund’s, CpG, Montanide, pluronic F-127	Mice; i.m.	[75]
Core	NP ^2^-PHB ^3^ in emulsion, CFA	Mice; s.c.	[76]
Core	ISCOMATRIX	Macaques; i.m.	[41]
E1E2, Core	ISCOMATRIX, MF59	Mice; i.m.	[41]
E1E2, Core	Alhydrogel	Mice; i.p.	[77]
E1E2; Polyprotein ^4^	ISCOMATRIX, MF59, CpG	Mice; i.m.	[78]
Polyprotein ^5^	Alhydrogel, MF59, CpG	Mice; i.m.	[79]
NS3 peptides	CAF09	Mice; i.p.	[80]
NS5A	MPL, NP ^2^-PEBEO ^6^	Mice; i.v.	[81]
NS3, NS5B	Pol-P ^7^, GMDP ^8^, IFN-α	Mice; s.c.	[82]
VLPs ^9^	AS01B, CpG	Mice; Baboons; i.m.	[83,84]
VLPs ^9^	Ahydrogel, E_8_Pam_2_Cys,	Mice; s.c.	[85]
VLPs ^9^	Alhydrogel, CFA	Mice	[86]
VLPs ^9^	Alhydrogel, CFA, Montanide	Mice, s.c.	[87]
VLPs ^10^	Alhydrogel, Montanide	Mice; i.m., i.n.	[88]
sE2 NP (Ferritin)	Alhydrogel, CpG	Mice; i.p.	[89]
NP-Polyprotein ^11^	PADRE ^12^, lipopeptide ^13^, IL-2	Mice; s.c.	[90]
HCV particles ^14^	MPL-trehalose	Mice; i.p.	[91]
	Alhydrogel; CpG-K3-SPG ^15^	Marmosets	[92]
E2 Core nanoparticles	Addavax	Mice; s.c.	[93]

^1^ Administration route: s.c.—subcutaneous; i.m.—intramuscular; i.p.—intraperitoneal; i.v.—intraveneous; i.n.—intranasal. ^2^ NP—nanoparticles. ^3^ PHB—polyhydroxybutyrate. ^4^ NS3, NS4, NS5. ^5^ Core, NS3, NS4a, NS4b, NS5a and NS5b. ^6^ PEBEO—poly((ethylene-co-butylene)-b-(ethylene oxide)). ^7^ Pol-P—polyprenyl phosphate -derivative of isoprenoids. ^8^ GMDP—glucosaminyl muramyl dipeptide. ^9^ Core, E1, E2. ^10^ C-terminal truncated core. ^11^ Polyyprotein—epitopes of NS3, NS4ab, and NS5a. ^12^ PADRE—pan HLA DR-binding epitope. ^13^ *Neisseria meningitidis*. ^14^ Inactivated; cell culture derived. ^15^ K3-SPG—Schizophyllan (polysaccharide).

**Table 2:** Delivery technologies for HCV DNA vaccine candidates.

Antigen	Adjuvants/Delivery	Animal Model Route ^1^	References
E2 plasmid	Electroporation; cytokine encoding plasmids	Mice	[98]
E2 plasmid	Electroporation	Mice, rats, rabbits; i.m.	[97]
E1E2 plasmid	Cationic PLGA microparticles	Mice, macaques	[71]
Core plasmid	Gold nanoparticles (electric pulses)	Mice; i.m.	[99]
Core plasmid	GM-CSF and IL-23	Mice; i.m.	[100]
NS3, NS4, NS5 plasmids	Plasmid encoding IL-28B, electroporation	Mice, i.m.,	[101]
NS3 plasmid	Hemolysin ^2^	Mice; i.d.	[102]
NS 3/4A plasmid	Elecroporation	Mice; i.m.	[103]
NS 3/4A plasmid	HBcAg gene sequence	Mice; i.m.	[104]
NS3 DNA ^3^	IL-12	Mice; i.m.	[105]

^1^ Administration route: s.c.—subcutaneous; i.m.—intramuscular; i.p.—intraperitoneal; i.v.—intraveneous; i.n.—intranasal. ^2^ Hemolysin—bacterial toxin, detoxified (Listeriolysin O). ^3^ Eukaryotic expression vector.

## Data Availability

Not applicable.

## References

[1] World Health Organization Hepatitis C. https://www.who.int/news-room/fact-sheets/detail/hepatitis-c.

[2] Cox A.L. (2015). Global control of hepatitis C virus. Science.

[3] Walker C.M., Grakoui A. (2015). Hepatitis C virus: Why do we need a vaccine to prevent a curable persistent infection?. Curr. Opin. Immunol..

[4] Pierce B.G., Keck Z.-Y., Foung S.K.H. (2016). Viral evasion and challenges of hepatitis C virus vaccine development. Curr. Opin. Virol..

[5] Bailey J.R., Barnes E., Cox A.L. (2019). Approaches, Progress, and Challenges to Hepatitis C Vaccine Development. Gastroenterology.

[6] Kinchen V.J., Cox A.L., Bailey J.R. (2018). Can broadly neutralizing monoclonal antibodies lead to a hepatitis C virus vaccine?. Trends Microbiol..

[7] Kinchen V.J., Zahid M.N., Flyak A.I., Soliman M.G., Learn G.H., Wang S., Davidson E., Doranz B.J., Ray S.C., Cox A.L. (2018). Broadly neutralizing antibody mediated clearance of human hepatitis C virus infection. Cell Host Microbe.

[8] Raghuraman S., Park H., Osburn W.O., Winkelstein E., Edlin B.R., Rehermann B. (2012). Spontaneous clearance of chronic hepatitis C virus infection is associated with appearance of neutralizing antibodies and reversal of T-cell exhaustion. J. Infect. Dis..

[9] Pestka J.M., Zeisel M.B., Bläser E., Schürmann P., Bartosch B., Cosset F.-L., Patel A.H., Meisel H., Baumert J., Viazov S. (2007). Rapid induction of virus-neutralizing antibodies and viral clearance in a single-source outbreak of hepatitis C. Proc. Natl. Acad. Sci. USA.

[10] Pashine A., Valiante N.M., Ulmer J.B. (2005). Targeting the innate immune response with improved vaccine adjuvants. Nat. Med..

[11] Liang T.J. (2013). Current progress in development of hepatitis C virus vaccines. Nat. Med..

[12] Rappuoli R., Pizza M., Del Giudice G., De Gregorio E. (2014). Vaccines, new opportunities for a new society. Proc. Natl. Acad. Sci. USA.

[13] De Gregorio E., Rappuoli R. (2014). From empiricism to rational design: A personal perspective of the evolution of vaccine development. Nat. Rev. Immunol..

[14] Reed S.G., Orr M.T., Fox C.B. (2013). Key roles of adjuvants in modern vaccines. Nat. Med..

[15] Nanishi E., Dowling D.J., Levy O. (2020). Toward precision adjuvants: Optimizing science and safety. Curr. Opin. Pediatr..

[16] Del Giudice G., Rappuoli R., Didierlaurent A.M. (2018). Correlates of adjuvanticity: A review on adjuvants in licensed vaccines. Semin. Immunol..

[17] Brito L.A., Malyala P., O’Hagan D.T. (2013). Vaccine adjuvant formulations: A pharmaceutical perspective. Semin. Immunol..

[18] Sepulveda-Crespo D., Resino S., Martinez I. (2020). Innate immune response against hepatitis C virus: Targets for vaccine adjuvants. Vaccines.

[19] Broz P., Monack D.M. (2013). Newly described pattern recognition receptors team up against intracellular pathogens. Nat. Rev. Immunol..

[20] Vasou A., Sultanoglu N., Goodbourn S., Randall R.E., Kostrikis L.G. (2017). Targeting pattern recognition receptors (PRR) for vaccine adjuvantation: From synthetic PRR agonists to the potential of defective interfering particles of viruses. Viruses.

[21] Olive C. (2012). Pattern recognition receptors: Sentinels in innate immunity and targets of new vaccine adjuvants. Expert Rev. Vaccines.

[22] HogenEsch H., O’Hagan D.T., Fox C.B. (2018). Optimizing the utilization of aluminum adjuvants in vaccines: You might just get what you want. npj Vaccines.

[23] De Gregorio E., Tritto E., Rappuoli R. (2008). Alum adjuvanticity: Unraveling a century old mystery. Eur. J. Immunol..

[24] Li H., Willingham S.B., Ting J.P.-Y., Re F. (2008). Cutting Edge: Inflammasome Activation by Alum and Alum’s Adjuvant Effect Are Mediated by NLRP3. J. Immunol..

[25] Lambrecht B.N., Kool M., Willart M.A.M., Hammad H. (2009). Mechanism of action of clinically approved adjuvants. Curr. Opin. Immunol..

[26] Stils H.F. (2005). Adjuvants and Antibody Production: Dispelling the Myths Associated with Freund’s Complete and Other Adjuvants. ILAR J..

[27] O’Hagan D.T., Ott G.S., Nest G.V., Rappuoli R., Giudice G.D. (2013). The history of MF59? adjuvant: A phoenix that arose from the ashes. Expert Rev. Vaccines.

[28] van Diepen M.T., Chapman R., Moore P.L., Margolin E., Hermanus T., Morris L., Ximba P., Rybicki E.P., Williamson A.-L. (2018). The adjuvant AlhydroGel elicits higher antibody titres than AddaVax when combined with HIV-1 subtype C gp140 from CAP256. PLoS ONE.

[29] van Doorn E., Liu H., Huckriede A., Hak E. (2016). Safety and tolerability evaluation of the use of Montanide ISA™51 as vaccine adjuvant: A systematic review. Hum. Vaccines Immunother..

[30] Jain S., O’Hagan D.T., Singh M. (2011). The long-term potential of biodegradable poly(lactideco-glycolide) microparticles as the next-generation vaccine adjuvant. Expert Rev. Vaccines.

[31] Andrianov A.K., Langer R. (2021). Polyphosphazene immunoadjuvants: Historical perspective and recent advances. J. Control. Release.

[32] Andrianov A.K., Marin A., Wang R., Karauzum H., Chowdhury A., Agnihotri P., Abdul S.Y., Mariuzza R.A., Fuerst T.R. (2020). Supramolecular assembly of Toll-like receptor 7/8 agonist into multimeric water-soluble constructs enables superior immune stimulation in vitro and in vivo. ACS Appl. Bio Mater..

[33] Andrianov A.K., Marin A., Fuerst T.R. (2016). Molecular-Level Interactions of Polyphosphazene Immunoadjuvants and Their Potential Role in Antigen Presentation and Cell Stimulation. Biomacromolecules.

[34] Andrianov A.K., Decollibus D.P., Marin A., Webb A., Griffin Y., Webby R.J. (2011). PCPP-formulated H5N1 influenza vaccine displays improved stability and dose-sparing effect in lethal challenge studies. J. Pharm. Sci..

[35] Palmer C.D., Ninković J., Prokopowicz Z.M., Mancuso C.J., Marin A., Andrianov A.K., Dowling D.J., Levy O. (2014). The effect of stable macromolecular complexes of ionic polyphosphazene on HIV Gag antigen and on activation of human dendritic cells and presentation to T-cells. Biomaterials.

[36] Marin A., DeCollibus D.P., Andrianov A.K. (2010). Protein Stabilization in Aqueous Solutions of Polyphosphazene Polyelectrolyte and Non-Ionic Surfactants. Biomacromolecules.

[37] Kabanov A., Zhu J., Alakhov V. (2005). Pluronic Block Copolymers for Gene Delivery. Adv. Genet..

[38] Coeshott C.M., Smithson S.L., Verderber E., Samaniego A., Blonder J.M., Rosenthal G.J., Westerink M.A.J. (2004). Pluronic^®^ F127-based systemic vaccine delivery systems. Vaccine.

[39] Kersten G.F.A., Crommelin D.J.A. (2003). Liposomes and ISCOMs. Vaccine.

[40] Pearse M.J., Drane D. (2005). ISCOMATRIX^®^ adjuvant for antigen delivery. Adv. Drug Delivery Rev..

[41] Polakos N.K., Drane D., Cox J., Ng P., Selby M.J., Chien D., O’Hagan D.T., Houghton M., Paliard X. (2001). Characterization of hepatitis C virus core-specific immune responses primed in rhesus macaques by a nonclassical ISCOM vaccine. J. Immunol..

[42] Schwendener R.A. (2014). Liposomes as vaccine delivery systems: A review of the recent advances. Ther. Adv. Vaccines.

[43] Didierlaurent A.M., Laupèze B., Di Pasquale A., Hergli N., Collignon C., Garçon N. (2017). Adjuvant system AS01: Helping to overcome the challenges of modern vaccines. Expert Rev. Vaccines.

[44] Agger E.M., Rosenkrands I., Hansen J., Brahimi K., Vandahl B.S., Aagaard C., Werninghaus K., Kirschning C., Lang R., Christensen D. (2008). Cationic liposomes formulated with synthetic mycobacterial cordfactor (CAF01): A versatile adjuvant for vaccines with different immunological requirements. PLoS ONE.

[45] Wegmann F., Moghaddam A.E., Schiffner T., Gartlan K.H., Powell T.J., Russell R.A., Baart M., Carrow E.W., Sattentau Q.J. (2015). The Carbomer-Lecithin Adjuvant Adjuplex Has Potent Immunoactivating Properties and Elicits Protective Adaptive Immunity against Influenza Virus Challenge in Mice. Clin. Vaccine Immunol..

[46] Bode C., Zhao G., Steinhagen F., Kinjo T., Klinman D.M. (2011). CpG DNA as a vaccine adjuvant. Expert Rev. Vaccines.

[47] Ciabattini A., Pettini E., Fiorino F., Pastore G., Andersen P., Pozzi G., Medaglini D. (2016). Modulation of Primary Immune Response by Different Vaccine Adjuvants. Front. Immunol..

[48] Campbell J.D. (2017). Development of the CpG Adjuvant 1018: A Case Study. Methods Mol. Biol..

[49] Lee G.-H., Lim S.-G. (2021). CpG-Adjuvanted Hepatitis B Vaccine (HEPLISAV-B^®^) Update. Expert Rev. Vaccines.

[50] Hemmi H., Kaisho T., Takeuchi O., Sato S., Sanjo H., Hoshino K., Horiuchi T., Tomizawa H., Takeda K., Akira S. (2002). Small anti-viral compounds activate immune cells via the TLR7 MyD88-dependent signaling pathway. Nat. Immunol..

[51] Jurk M., Heil F., Vollmer J., Schetter C., Krieg A.M., Wagner H., Lipford G., Bauer S. (2002). Human TLR7 or TLR8 independently confer responsiveness to the antiviral compound R-848. Nat. Immunol..

[52] Schön M.P., Schön M. (2008). TLR7 and TLR8 as targets in cancer therapy. Oncogene.

[53] Weeratna R.D., Makinen S.R., McCluskie M.J., Davis H.L. (2005). TLR agonists as vaccine adjuvants: Comparison of CpG ODN and Resiquimod (R-848). Vaccine.

[54] Tomai M.A., Miller R.L., Lipson K.E., Kieper W.C., Zarraga I.E., Vasilakos J.P. (2007). Resiquimod and other immune response modifiers as vaccine adjuvants. Expert Rev. Vaccines.

[55] Tomai M.A., Vasilakos J.P. (2011). TLR-7 and -8 agonists as vaccine adjuvants. Expert Rev. Vaccines.

[56] Jacobsen N.E., Fairbrother W.J., Kensil C.R., Lim A., Wheeler D.A., Powell M.F. (1996). Structure of the saponin adjuvant QS-21 and its base-catalyzed isomerization product by 1H and natural abundance 13C NMR spectroscopy. Carbohydr Res..

[57] Lacaille-Dubois M.-A. (2019). Updated insights into the mechanism of action and clinical profile of the immunoadjuvant QS-21: A review. Phytomedicine.

[58] Marty-Roix R., Vladimer G.I., Pouliot K., Weng D., Buglione-Corbett R., West K., MacMicking J.D., Chee J.D., Wang S., Lu S. (2016). Identification of QS-21 as an Inflammasome-activating Molecular Component of Saponin Adjuvants. J. Biol. Chem..

[59] Matsumoto M., Seya T. (2008). TLR3: Interferon induction by double-stranded RNA including poly(I:C). Adv. Drug Delivery Rev..

[60] Jackson D.C., Lau Y.F., Le T., Suhrbier A., Deliyannis G., Cheers C., Smith C., Zeng W., Brown L.E. (2004). A totally synthetic vaccine of generic structure that targets Toll-like receptor 2 on dendritic cells and promotes antibody or cytotoxic T cell responses. Proc. Natl. Acad. Sci. USA.

[61] Chua B.Y., Pejoski D., Turner S.J., Zeng W., Jackson D.C. (2011). Soluble proteins induce strong CD8+ T cell and antibody responses through electrostatic association with simple cationic or anionic lipopeptides that target TLR2. J. Immunol..

[62] Škrnjug I., Rueckert C., Libanova R., Lienenklaus S., Weiss S., Guzmán C.A. (2014). The Mucosal Adjuvant Cyclic di-AMP Exerts Immune Stimulatory Effects on Dendritic Cells and Macrophages. PLoS ONE.

[63] Volckmar J., Knop L., Stegemann-Koniszewski S., Schulze K., Ebensen T., Guzmán C.A., Bruder D. (2019). The STING activator c-di-AMP exerts superior adjuvant properties than the formulation poly(I:C)/CpG after subcutaneous vaccination with soluble protein antigen or DEC-205-mediated antigen targeting to dendritic cells. Vaccine.

[64] Ishikawa H., Barber G.N. (2008). STING is an endoplasmic reticulum adaptor that facilitates innate immune signalling. Nature.

[65] Bazzill J.D., Ochyl L.J., Giang E., Castillo S., Law M., Moon J.J. (2018). Interrogation of Antigen Display on Individual Vaccine Nanoparticles for Achieving Neutralizing Antibody Responses against Hepatitis C Virus. Nano Lett..

[66] Andrianov A.K., Marin A., Wang R., Chowdhury A., Agnihotri P., Yunus A.S., Pierce B.G., Mariuzza R.A., Fuerst T.R. (2021). In Vivo and In Vitro Potency of Polyphosphazene Immunoadjuvants with Hepatitis C Virus Antigen and the Role of Their Supramolecular Assembly. Mol. Pharm..

[67] Pierce B.G., Keck Z.-Y., Wang R., Lau P., Garagusi K., Elkholy K., Toth E.A., Urbanowicz R.A., Guest J.D., Agnihotri P. (2020). Structure-based design of hepatitis C virus E2 glycoprotein improves serum binding and cross-neutralization. J. Virol..

[68] Center R.J., Boo I., Phu L., McGregor J., Poumbourios P., Drummer H.E. (2020). Enhancing the antigenicity and immunogenicity of monomeric forms of hepatitis C virus E2 for use as a preventive vaccine. J. Biol. Chem..

[69] Liu J., Feng X., Chen Z., Yang X., Shen Z., Guo M., Deng F., Liu Y., Zhang H., Chen C. (2019). The adjuvant effect of C60(OH)22 nanoparticles promoting both humoral and cellular immune responses to HCV recombinant proteins. Mater. Sci. Eng..

[70] Choo Q.L., Kuo G., Ralston R., Weiner A., Chien D., Van Nest G., Han J., Berger K., Thudium K., Kuo C. (1994). Vaccination of chimpanzees against infection by the hepatitis C virus. Proc. Natl. Acad. Sci. USA.

[71] O’Hagan D.T., Singh M., Dong C., Ugozzoli M., Berger K., Glazer E., Selby M., Wininger M., Ng P., Crawford K. (2004). Cationic microparticles are a potent delivery system for a HCV DNA vaccine. Vaccine.

[72] Chen F., Nagy K., Chavez D., Willis S., McBride R., Giang E., Honda A., Bukh J., Ordoukhanian P., Zhu J. (2020). Antibody Responses to Immunization With HCV Envelope Glycoproteins as a Baseline for B-Cell-Based Vaccine Development. Gastroenterology.

[73] Landi A., Law J., Hockman D., Logan M., Crawford K., Chen C., Kundu J., Ebensen T., Guzman C.A., Deschatelets L. (2017). Superior immunogenicity of HCV envelope glycoproteins when adjuvanted with cyclic-di-AMP, a STING activator or archaeosomes. Vaccine.

[74] Guest J.D., Wang R., Elkholy K.H., Chagas A., Chao K.L., Cleveland T.E., Kim Y.C., Keck Z.-Y., Marin A., Yunus A.S. (2021). Design of a native-like secreted form of the hepatitis C virus E1E2 heterodimer. Proc. Natl. Acad. Sci. USA.

[75] Roohvand F., Aghasadeghi M.-R., Sadat S.M., Budkowska A., Khabiri A.-R. (2007). HCV core protein immunization with Montanide/CpG elicits strong Th1/Th2 and long-lived CTL responses. Biochem. Biophys. Res. Commun..

[76] Parlane N.A., Grage K., Lee J.W., Buddle B.M., Denis M., Rehm B.H.A. (2011). Production of a Particulate Hepatitis C Vaccine Candidate by an Engineered *Lactococcus lactis* Strain. Appl. Environ. Microbiol..

[77] Martínez-Donato G., Musacchio A., Alvarez-Lajonchere L., Acosta-Rivero N., Amador Y., Guerra I., Peña D., Pérez A., Castro J., Puentes P. (2010). Ratio of HCV structural antigens in protein-based vaccine formulations is critical for functional immune response induction. Biotechnol. Appl. Biochem..

[78] Lin Y., Kwon T., Polo J., Zhu Y.F., Coates S., Crawford K., Dong C., Wininger M., Hall J., Selby M. (2008). Induction of broad CD4+ and CD8+ T-cell responses and cross-neutralizing antibodies against hepatitis C virus by vaccination with Th1-adjuvanted polypeptides followed by defective alphaviral particles expressing envelope glycoproteins gpE1 and gpE2 and nonstructural proteins 3, 4, and 5. J. Virol..

[79] Vajdy M., Selby M., Medina-Selby A., Coit D., Hall J., Tandeske L., Chien D., Hu C., Rosa D., Singh M. (2006). Hepatitis C virus polyprotein vaccine formulations capable of inducing broad antibody and cellular immune responses. J. Gen. Virol..

[80] Filskov J., Mikkelsen M., Hansen P.R., Christensen J.P., Thomsen A.R., Andersen P., Bukh J., Agger E.M. (2017). Broadening CD4+ and CD8+ T Cell Responses against Hepatitis C Virus by Vaccination with NS3 Overlapping Peptide Panels in Cross-Priming Liposomes. J. Virol..

[81] Fichter M., Piradashvili K., Pietrzak-Nguyen A., Pretsch L., Kuhn G., Strand S., Knuf M., Zepp F., Wurm F.R., Mailänder V. (2016). Polymeric hepatitis C virus non-structural protein 5A nanocapsules induce intrahepatic antigen-specific immune responses. Biomaterials.

[82] Masalova O.V., Lesnova E.I., Onishchuk A.A., Ivanova A.M., Gerasimova E.V., Ivanov A.V., Narovlyansky A.N., Sanin A.V., Pronin A.V., Kushch A.A. (2018). Polyprenyl Phosphates Induce a High Humoral and Cellular Response to Immunization with Recombinant Proteins of the Replicative Complex of the Hepatitis C Virus. Dokl. Biochem. Biophys..

[83] Qiao M., Murata K., Davis A.R., Jeong S.-H., Liang T.J. (2003). Hepatitis C virus–like particles combined with novel adjuvant systems enhance virus-specific immune responses. Hepatology.

[84] Jeong S.-H., Qiao M., Nascimbeni M., Hu Z., Rehermann B., Murthy K., Liang T.J. (2004). Immunization with Hepatitis C Virus-Like Particles Induces Humoral and Cellular Immune Responses in Nonhuman Primates. J. Virol..

[85] Chua B.Y., Johnson D., Tan A., Earnest-Silveira L., Sekiya T., Chin R., Torresi J., Jackson D.C. (2012). Hepatitis C VLPs Delivered to Dendritic Cells by a TLR2 Targeting Lipopeptide Results in Enhanced Antibody and Cell-Mediated Responses. PLoS ONE.

[86] Earnest-Silveira L., Chua B., Chin R., Christiansen D., Johnson D., Herrmann S., Ralph S.A., Vercauteren K., Mesalam A., Meuleman P. (2016). Characterization of a hepatitis C virus-like particle vaccine produced in a human hepatocyte-derived cell line. J. Gen. Virol..

[87] Christiansen D., Earnest-Silveira L., Chua B., Meuleman P., Boo I., Grubor-Bauk B., Jackson D.C., Keck Z.Y., Foung S.K.H., Drummer H.E. (2018). Immunological responses following administration of a genotype 1a/1b/2/3a quadrivalent HCV VLP vaccine. Sci. Rep..

[88] Acosta-Rivero N., Poutou J., Álvarez-Lajonchere L., Guerra I., Aguilera Y., Musacchio A., Rodríguez A., Aguilar J.C., Falcon V., Álvarez-Obregon J.C. (2009). Recombinant in vitro assembled hepatitis C virus core particles induce strong specific immunity enhanced by formulation with an oil-based adjuvant. Biol. Res..

[89] Yan Y., Wang X., Lou P., Hu Z., Qu P., Li D., Li Q., Xu Y., Niu J., He Y. (2020). A Nanoparticle-Based Hepatitis C Virus Vaccine With Enhanced Potency. J. Infect. Dis..

[90] Kuprianov V.V., Nikolaeva L.I., Zykova A.A., Dedova A.V., Grishechkin A.E., Kapustin I.V., Kotlyarov R.Y., Ravin N.V. (2020). Combination of three adjuvants enhances the immunogenicity of a recombinant protein containing the CTL epitopes of non-structural proteins of hepatitis C virus. Virus Res..

[91] Akazawa D., Moriyama M., Yokokawa H., Omi N., Watanabe N., Date T., Morikawa K., Aizaki H., Ishii K., Kato T. (2013). Neutralizing Antibodies Induced by Cell Culture–Derived Hepatitis C Virus Protect Against Infection in Mice. Gastroenterology.

[92] Yokokawa H., Higashino A., Suzuki S., Moriyama M., Nakamura N., Suzuki T., Suzuki R., Ishii K., Kobiyama K., Ishii K.J. (2018). Induction of humoural and cellular immunity by immunisation with HCV particle vaccine in a non-human primate model. Gut.

[93] He L., Tzarum N., Lin X., Shapero B., Sou C., Mann C.J., Stano A., Zhang L., Nagy K., Giang E. (2020). Proof of concept for rational design of hepatitis C virus E2 core nanoparticle vaccines. Sci. Adv..

[94] Giang E., Dorner M., Prentoe J.C., Dreux M., Evans M.J., Bukh J., Rice C.M., Ploss A., Burton D.R., Law M. (2012). Human broadly neutralizing antibodies to the envelope glycoprotein complex of hepatitis C virus. Proc. Natl. Acad. Sci. USA.

[95] Bartosch B., Dubuisson J., Cosset F.-L. (2003). Infectious hepatitis C virus pseudo-particles containing functional E1–E2 envelope protein complexes. J. Ex. Med..

[96] Hobernik D., Bros M. (2018). DNA vaccines—how far from clinical use?. Int. J. Mol. Sci..

[97] Zucchelli S., Capone S., Fattori E., Folgori A., Di Marco A., Casimiro D., Simon A.J., Laufer R., La Monica N., Cortese R. (2000). Enhancing B- and T-Cell Immune Response to a Hepatitis C Virus E2 DNA Vaccine by Intramuscular Electrical Gene Transfer. J. Virol..

[98] Arcuri M., Cappelletti M., Zampaglione I., Aurisicchio L., Nicosia A., Ciliberto G., Fattori E. (2008). Synergistic effect of gene-electro transfer and adjuvant cytokines in increasing the potency of hepatitis C virus genetic vaccination. J. Gene Med..

[99] Draz M.S., Wang Y.-J., Chen F.F., Xu Y., Shafiee H. (2017). Electrically Oscillating Plasmonic Nanoparticles for Enhanced DNA Vaccination against Hepatitis C Virus. Adv. Funct. Mater..

[100] Hartoonian C., Ebtekar M., Soleimanjahi H., Karami A., Mahdavi M., Rastgoo N., Azadmanesh K. (2009). Effect of immunological adjuvants: GM-CSF (granulocyte-monocyte colony stimulating factor) and IL-23 (interleukin-23) on immune responses generated against hepatitis C virus core DNA vaccine. Cytokine.

[101] Lee H., Jeong M., Oh J., Cho Y., Shen X., Stone J., Yan J., Rothkopf Z., Khan A.S., Cho B.M. (2017). Preclinical evaluation of multi antigenic HCV DNA vaccine for the prevention of Hepatitis C virus infection. Sci. Rep..

[102] Pouriayevali M.H., Bamdad T., Sadat S.M., Sadeghi S.A., Sabahi F., Mahdavi M., Aghasadeghi M.R. (2019). Listeriolysin O immunogenetic adjuvant enhanced potency of hepatitis C virus NS3 DNA vaccine. IUBMB Life.

[103] Ahlén G., Söderholm J., Tjelle T., Kjeken R., Frelin L., Höglund U., Blomberg P., Fons M., Mathiesen I., Sällberg M. (2007). In Vivo Electroporation Enhances the Immunogenicity of Hepatitis C Virus Nonstructural 3/4A DNA by Increased Local DNA Uptake, Protein Expression, Inflammation, and Infiltration of CD3^+^ T Cells. J. Immunol..

[104] Levander S., Sällberg M., Ahlén G., Frelin L. (2016). A non-human hepadnaviral adjuvant for hepatitis C virus-based genetic vaccines. Vaccine.

[105] Naderi M., Saeedi A., Moradi A., Kleshadi M., Zolfaghari M.R., Gorji A., Ghaemi A. (2013). Interleukin-12 as a genetic adjuvant enhances hepatitis C virus NS3 DNA vaccine immunogenicity. Virol. Sin..

[106] Ray R., Meyer K., Banerjee A., Basu A., Coates S., Abrignani S., Houghton M., Frey S.E., Belshe R.B. (2010). Characterization of antibodies induced by vaccination with hepatitis C virus envelope glycoproteins. J. Infect. Dis..

[107] Frey S.E., Houghton M., Coates S., Abrignani S., Chien D., Rosa D., Pileri P., Ray R., Di Bisceglie A.M., Rinella P. (2010). Safety and immunogenicity of HCV E1E2 vaccine adjuvanted with MF59 administered to healthy adults. Vaccine.

[108] Stamataki Z., Coates S., Abrignani S., Houghton M., McKeating J.A. (2011). Immunization of Human Volunteers With Hepatitis C Virus Envelope Glycoproteins Elicits Antibodies That Cross-Neutralize Heterologous Virus Strains. J. Infect. Dis..

[109] Colombatto P., Brunetto M.R., Maina A.M., Romagnoli V., Almasio P., Rumi M.G., Ascione A., Pinzello G., Mondelli M., Muratori L. (2014). HCV E1E2-MF59 vaccine in chronic hepatitis C patients treated with PEG-IFNα2a and Ribavirin: A randomized controlled trial. J. Viral Hepatitis.

[110] Law J.L.M., Chen C., Wong J., Hockman D., Santer D.M., Frey S.E., Belshe R.B., Wakita T., Bukh J., Jones C.T. (2013). A Hepatitis C Virus (HCV) Vaccine Comprising Envelope Glycoproteins gpE1/gpE2 Derived from a Single Isolate Elicits Broad Cross-Genotype Neutralizing Antibodies in Humans. PLoS ONE.

[111] Leroux-Roels G., Depla E., Hulstaert F., Tobback L., Dincq S., Desmet J., Desombere I., Maertens G. (2004). A candidate vaccine based on the hepatitis C E1 protein: Tolerability and immunogenicity in healthy volunteers. Vaccine.

[112] Leroux-Roels G., Batens A.-H., Desombere I., Steen B.V.D., Stichele C.V., Maertens G., Hulstaert F. (2005). Immunogenicity and Tolerability of Intradermal Administration of an HCV E1-Based Vaccine Candidate in Healthy Volunteers and Patients with Resolved or Ongoing Chronic HCV Infection. Hum. Vaccines.

[113] Drane D., Maraskovsky E., Gibson R., Mitchell S., Barnden M., Moskwa A., Shaw D., Gervase B., Coates S., Houghton M. (2009). Priming of CD4+ and CD8+ T cell responses using a HCV core ISCOMATRIX™ vaccine: A phase I study in healthy volunteers. Hum. Vaccines.

[114] Firbas C., Jilma B., Tauber E., Buerger V., Jelovcan S., Lingnau K., Buschle M., Frisch J., Klade C.S. (2006). Immunogenicity and safety of a novel therapeutic hepatitis C virus (HCV) peptide vaccine: A randomized, placebo controlled trial for dose optimization in 128 healthy subjects. Vaccine.

[115] Klade C.S., Wedemeyer H., Berg T., Hinrichsen H., Cholewinska G., Zeuzem S., Blum H., Buschle M., Jelovcan S., Buerger V. (2008). Therapeutic Vaccination of Chronic Hepatitis C Nonresponder Patients With the Peptide Vaccine IC41. Gastroenterology.

[116] Firbas C., Boehm T., Buerger V., Schuller E., Sabarth N., Jilma B., Klade C.S. (2010). Immunogenicity and safety of different injection routes and schedules of IC41, a Hepatitis C virus (HCV) peptide vaccine. Vaccine.

[117] Han J.W., Sung P.S., Hong S.-H., Lee H., Koh J.Y., Lee H., White S., Maslow J.N., Weiner D.B., Park S.-H. (2020). IFNL3-adjuvanted HCV DNA vaccine reduces regulatory T cell frequency and increases virus-specific T cell responses. J. Hepatol..

